# Digital Person-Centered Self-Management Support for People With Type 2 Diabetes: Qualitative Study Exploring Design Challenges

**DOI:** 10.2196/10702

**Published:** 2019-09-19

**Authors:** Robyn Schimmer, Carljohan Orre, Ulrika Öberg, Karin Danielsson, Åsa Hörnsten

**Affiliations:** 1 Department of Informatics Umeå University Umeå Sweden; 2 Department of Computer Science and Media Technology Malmö University Malmö Sweden; 3 Department of Nursing Umeå University Umeå Sweden

**Keywords:** eHealth, diabetes mellitus, type 2, informatics, nursing, patient-centered care, self-management

## Abstract

**Background:**

Self-management is a substantial part of treatment for patients with type 2 diabetes (T2D). Modern digital technology, being small, available, and ubiquitous, might work well in supporting self-management. This study follows the process of developing a pilot implementation of an electronic health (eHealth) service for T2D self-management support in primary health care. The use of digital health, or eHealth, solutions for supporting self-management for patients with T2D is increasing. There are good examples of successful implementations that can serve as guides in the development of new solutions. However, when adding person-centered principles as a requirement, the examples are scarce.

**Objective:**

The objective of this study was to explore challenges that could impact the design of a person-centered eHealth service for T2D self-management support. The study included data collection from multiple sources, that is, interviews, observations, focus groups, and a Mentimeter (interactive presentation with polling) survey among stakeholders, representing various perspectives of T2D.

**Methods:**

A user-centered design approach was used to exploratively collect data from different sources. Data were collected from a workshop, interviews, and observations. The different data sources enabled a triangulation of data.

**Results:**

Results show that user needs related to an eHealth service for person-centered T2D self-management support are multifaceted and situated in a complex context. The two main user groups, patients and diabetes specialist nurses, express needs that both diverge and converge, which indicates that critical design decisions have to be made. There is also a discrepancy between the needs expressed by the potential users and the current work practice, suggesting more attention toward changing the organization of work to fully support a new eHealth service.

**Conclusions:**

A total of three overarching challenges—flexible access, reducing administrative tasks, and patient empowerment—each having a significant impact on design, are discussed. These challenges need to be considered and resolved through careful design decisions. Special attention has to be given to the patient user group that could greatly impact current work practice and power structures at the primary care unit. A need for further studies investigating patient needs in everyday life is identified to better support the implementation of technology that does not give specific attention to organizational perspectives but instead approach design with the patient perspective in focus.

## Introduction

### Background

Using electronic health (eHealth) services as self-management support for people with type 2 diabetes (T2D) is, in many ways, a promising way to reduce costs, increase availability of care, and empower patients [1-3]. T2D is a common diagnosis and demands a high level of self-management. In Sweden, 4% to 6% of the population is estimated to suffer from diabetes, although approximately 4% is diagnosed and whereof approximately 90% is T2D [4,5]. However, as most people are diagnosed at an age of 60 years and above, the prevalence of T2D is much higher in the older age groups [6]. In the group of people aged 65 years and older, the prevalence is reported to be approximately 12% to 18%, with higher percentage among men [7]. The illness is complex and demanding as the basic treatment is dietary changes and increased physical activity besides the pharmaceutical treatment and blood sugar testing. It also commonly involves comorbidities such as hypertension, hyperlipidemia, and obesity and leads to severe complications such as stroke and heart disease, kidney dysfunction, blindness, and other problems if not sufficiently treated and self-managed by patients [8].

The context of this paper is Swedish primary health care, which is responsible for treating people with T2D. Basic medical treatment as well as nursing, prevention, and rehabilitation that do not demand special competences are offered in primary health care. Patients with chronic illnesses as T2D visit the primary health care center on a regular basis. General practitioners, primary health care nurses with special responsibility for diabetes clinics, physiotherapists, and occupational therapists often work in teams at primary health care centers. Majority of them are connected to the public health welfare program.

Studies have shown that patients see a potential in using eHealth services to support self-management [9,10]. The development of such eHealth services has been ongoing for many years and with various outcomes. In a systematic review from 2013, Pal et al [11] show that although internet-based interventions for diabetes self-management have limited effects on glycemic control, mobile phone–based interventions demonstrated more promising results. In a more recent study, Murray et al [12] report improvements in glycemic control through a Web-based self-management program.

The variety of outcomes reported in different studies implies a challenge in how eHealth services for self-management support should be designed, implemented, and evaluated. Reviewing the supply of available apps for diabetes management, Huang et al [13] conclude that “apps could play an important role in complementing multifaceted diabetes care” and also highlight the importance of being context-specific and adaptive to specific user’s needs. When addressing, for example, specific context and user needs, it is important to clearly describe how these perspectives are applied in design and implementation processes. Discussing the potential of self-management eHealth interventions for social support, Vorderstrasse et al [14] conclude that many studies lack detailed descriptions about how social support has been designed, implemented, and evaluated. It is, therefore, hard to determine the factors that have impact on social support.

### Person-Centered Care and Electronic Health

The American Diabetes Association recommends [8] that person-centered care (PCC) approaches should be applied in self-management support. There are benefits of adding PCC functionality to eHealth services, both for patients and health care organizations [15]. Digital devices that capture personal data and behaviors can be utilized to develop more personalized and timely services [16]. However, going through current literature, there are still few examples of eHealth services for T2D self-management support that also incorporate PCC. Wildevuur et al [17] present a set of preconditions for information and communications technology–enabled PCC and also conclude that this is a relatively new research area. They also point out the need for more research on design of technology that integrates a person-centered approach with attention to the context of use and user experience.

In this paper, we address these challenges related to design and technology for PCC. Design of eHealth services, as with all information technology (IT), is not a matter of solving simple problems but rather finding a possible composition that meets the requirements. Exploring challenges faced in a design situation of an eHealth service that includes PCC principles is, therefore, crucial for future work integrating them into the design of new eHealth services. The study is a part of our work with an ongoing pilot implementation study to develop a person-centered eHealth service for T2D self-management support. The aim of this study was to explore possible challenges that could impact the design of a person-centered eHealth service for T2D self-management support in Swedish primary health care.

## Methods

### General Approach

This particular study is part of a larger project with the aim of exploring the prerequisites for and developing a person-centered eHealth service for support of self-management in T2D. The project has already published several part studies [10,18,19]. In this study, data are collected from multiple sources, that is, interviews, observations, focus groups, and a Mentimeter (interactive presentation with polling) survey among stakeholders, representing various perspectives of T2D. Identifying design challenges requires a broad approach where user requirements and needs are supplemented with an understanding of the context of use, current organization, and work practice. The choice of data sources for this study was made with this systemic view in mind, where each data source provided insights into different aspects of the whole.

### User-Centered Design

Due to the focus on PCC, we wanted to move beyond a solely health care perspective where organizational needs are prioritized. Instead, we wanted to have a more holistic viewpoint where the patients and their needs were foremost. We, therefore, chose to adopt a user-centered design (UCD) approach. The strength of the user-centered approach is that it is founded on the principle of designing based on studying user’s practice [20], which fits well with the purpose of adopting person-centered principles into the eHealth service. A design approach is also well suited for exploring complex problems [21] and context of use [22,23].

UCD can beneficially be used to focus on important aspects such as *multiple stakeholders*, *current practice*, and *future needs* [20,24,25]. These three aspects formed the foundation for data collection where different data sources were used to gather data that helped us explore these aspects.

#### Multiple Stakeholders

Understanding the users and other stakeholders is an essential part of design [26]. A complex context often includes multiple users and stakeholders that require to be assessed to identify user needs and organizational constraints.

The two main user groups identified for this study were patients with T2D and diabetes specialist nurses. These two groups are the main actors in most of the current Swedish primary health care practice. As most patients manage their own health in everyday life, with help and support from family members, it makes them important stakeholders too. Beyond these groups, there are also other user groups and stakeholders involved such as managers and physicians at the primary care unit and representatives from higher organizational levels. However, no stakeholders on higher organizational levels than the regional primary care director were participating in our studies. Furthermore, researchers and system developers were also seen as stakeholders important for the design and development of eHealth service.

#### Current Practice

Understanding the context of use is an important part of the design process. With the patient and the diabetes specialist nurse as the two main user groups, there are two distinctly different contexts of use: one situated in the patients’ everyday life and the other that is situated at the primary care unit and the work of the diabetes specialist nurse. Together, they form a complex context that is necessary to understand to design an eHealth service that is adapted for the individual’s use in everyday life; nurses’ caring, treatment, and administrative aspects of work; and interaction between these two user groups.

Current practice is an aspect related to the context and focuses on the processes and activities in the current situation. For the design process, exploring current practice is an important part of making decisions, whether certain activities should be supported by the new system or whether it is necessary to make adjustments in the routines [27]. Within this context, current practice involves activities of the individual in everyday life and activities more closely related to the processes at the primary care unit. For this study, we choose to focus primarily on the primary care unit context and the interactions between patients and diabetes specialist nurses. The assumption was that these interactions between patients and nurses were likely to reveal challenges and tensions that were important in guiding design decisions.

#### Future Needs

Design involves creating something that is not yet there [28]. An important part of design is, therefore, to establish the users’ future needs. Identifying future needs is done through careful investigations into the current work practice and requirements expressed by future users. Users are, however, often limited in their ability to fully express what a new system should include [29].

Understanding and establishing future needs is not only an empirical inquiry into current practice and contexts that connects requirements and wishes expressed by future users but also an analytical activity where the results of the inquiry are analyzed in relation to available technology and other factors. Together with other relevant material, future needs form the foundation for important design decisions [23,28] by setting out the desired direction for the new design.

### Data Collection and Participants

Data collection from multiple sources, that is, interviews, observations, focus groups, and a Mentimeter survey among stakeholders, representing various perspectives of T2D, was performed.

### Interviews and Observations

Study participants for the interviews and observations were recruited through contacting the regional primary care director who appointed 1 health care center as a possible choice. The manager of this health care center accepted their participation and mediated contact with their diabetes nurses. The manager and 1 diabetes nurse both had many years of work experience within primary health care and T2D care. All interviews and observations were made by the first author.

Repeated interviews (n=4) with 1 diabetes specialist nurse and 1 primary care unit manager (n=2) revolved around work process, routines, and known organizational constraints and challenges. The purpose of this data collection was to gain insights into opportunities and constraints related to the organization and professional self-management support. All interviews were audio recorded and transcribed verbatim for analysis.

Nonparticipatory observations (n=4) of nurse-patient consultations were conducted at the health care center using video (GoPro cameras) to record the sessions. The purpose of the observations was to gain a deeper understanding of the preconditions for interaction between the diabetes specialist nurse and the patient.

### Workshop Procedures

Informed by the interviews and observations and to address the multifaceted perspectives of multiple stakeholders, we chose to design a workshop where we invited a broad sample of stakeholders somehow involved in T2D care. The recruitment began by listing potentially important stakeholders and participants who were invited based on personal contacts and snowball sampling. The purpose of the workshop that was held in 2016 was to explore expectations, wishes and needs, and concerns related to self-management support in T2D care and digitalization. The list of participants is presented in Table 1.

**Table 1:** Workshop participants (N=26).

Participant	Value, n (%)
Patients	5 (19)
Spouses	2 (8)
Diabetes specialist nurses	9 (35)
Physician and regional primary care director	1 (4)
Researchers	6 (23)
System developers	3 (12)

### Focus Groups and Mentimeter Surveys

For focus group discussions during the workshop, the participants (n=26) were divided into 4 focus groups of 6 to 7 people. Participants were divided so that there were 1 to 2 patients and/or spouses per group and 2 to 3 diabetes specialist nurses per group. The other participants, including researchers, were distributed evenly into the 4 groups. The idea of using mixed groups was to let different perspectives be expressed and shared to explore both mutual and divergent expectations and opinions.

To support group discussions, each focus group worked with two canvases. On the basis of the idea of business canvases [30], these were designed to cover different aspects of self-management and digitalization. Additional material, multicolored post-it notes, pens, and markers were available and used by the participants to document the discussions.

Mentimeter questions had been prepared to cover questions on concept definitions (eg, *what does self-management mean to you?*) and user technology. The participants used smartphones or tablets to answer the questions individually, and the answers could then be presented anonymously to further inspire group discussions.

### Analysis

Nonparticipatory observations of nurse-patient consultations were conducted using video to record the sessions—focus lays on preconditions for and outcome of interaction between the diabetes specialist nurse and the patient. Video recordings from the observations were viewed in sequence, using memos to annotate important activities and situations [31]. The recordings and memos were then discussed, analyzed, and interpreted by the authors. Themes (*increased use of checklists, avoiding one-sided communication,* and *prioritizing among administrative tasks*) were identified, which suggested that changes were needed to make patients more involved.

Repeated interviews, conducted individually and in pair, were performed with 1 diabetes specialist nurse and 1 primary care unit manager. Interview guides were used that covered areas such as work processes, routines, and organizational constraints and challenges for T2D care. The purpose of this data collection was to gain insights on opportunities and constraints related to the organization and routines in care and care processes. All interviews were audio recorded and transcribed verbatim for analysis.

All text data were then analyzed using qualitative content analysis [32]. The texts were read through several times, and meaning units responding to the aim were identified in the interview data. In the next step, all texts in meaning units were coded and organized based on similarities and dissimilarities. The codes were sorted and abstracted into themes, illustrating emergent concerns expressed by the participants. Data on various levels were discussed between the authors to gain consensus and reach trustworthiness regarding the interpreted themes (*access governed by needs, developed teamwork, relevant IT training, assessing patients’ individual needs,* and *counteracting shallow patient interaction).*

Focus group discussions in 4 groups were conducted during the workshop (2×45 min). The first session revolved around personal needs and possible improvements related to both everyday life and care. Questions raised were as follows: needs that are not satisfied today, what patients and spouses expect from care, and possible future improvements. The second sessions revolved around perceptions and expectations on eHealth and digital self-management support. Questions raised were as follows: negative expectations and fears toward digitalization, advantages with digitalization, and digital solutions and functions that could improve life with diabetes. Data from focus group discussions comprised canvases, multicolored post-it notes, pens, and markers that were used by the participants for self-documentation that was analyzed through systematic text condensation [33]. In addition, field notes made by the first author who moved around in the room observing and listening to all focus groups were analyzed together with the self-documented data (*striving for disease control and balanced life, practicing PCC, facing limited resources,* and *increasing number of contact channels*).

Mentimeter questions had been prepared in advance to cover questions related to concept definitions (eg, *what does self-management mean to you?* and *what does eHealth mean to you?*). Using smartphones or tablets, the participants answered the questions, and the results were displayed collectively (without revealing individual respondents). The purpose was to further inspire the discussions and create common understanding of key concepts. The data collected were added to the focus group discussion dataset and field notes for analysis. The analyzed Mentimeter survey data were about self-management (*improved responsibility and self-care, lifelong learning needs,* and *promoting independency*).

Data from all data collections were discussed and analyzed between the authors. Qualitative content analysis was used to sort data on various levels into 7 themes (*improved access, resource efficiency, reduced administrative tasks, continuous training and support, tailored care, strengthened communication,* and *patient empowerment*).

### Ethics

Ethical approval was given by the regional ethics board in Umeå for all patient involvement (Registration number 2016/375-32). All patients were informed about the aim of the study and accepted participation in the study and expressed no doubts.

## Results

### Themes and Subthemes

The aim of this study was to explore challenges that could impact the design of a person-centered eHealth service for T2D self-management support. The design process included data collection from multiple sources, for example, interviews, observations, focus groups, and Mentimeter survey among stakeholders, representing various perspectives of T2D.

The analysis revealed 7 critical factors, or challenges, expressed as the following themes: improved access, resource efficiency, reduced administrative tasks, continuous training and support, tailored care, strengthened communication, and finally, patient empowerment (Table 2).

### Improved Access

Getting in contact with the caregiver was an emergent theme among the patients participating in the workshop. They requested increased number of contact channels to establish contact with the caregiver in an easier and quicker way. One of the patients expressed that it should be “Easy to get in contact, e.g., getting advice and new prescriptions.” Questions or issues could emerge at any given time, and it is not always meaningful to wait to raise this issue, for example, acute hypoglycemia, until the next regular meeting with the nurse or the physician (which could be 6 months away). Even if the issue is of temporal importance, getting a quick answer and support could strengthen the overall service experience and support patient learning.

This is, however, not reflected in what was seen at the primary care unit; there was no simple way for patients to interact directly with their diabetes specialist nurse without going through the generic booking or contact routine, as access must be governed by needs. This routine comprises contacting the primary care unit reception either by phone or using the existing Web-based system for bookings or requests. The request is evaluated and redirected to someone at the primary care unit that is considered appropriate (or has the time) to answer the request. Then, the patient is contacted later over phone, which could lead to a discrepancy between the urgency of the issue and the time of being contacted.

### Resource Efficiency

Being efficient with limited resources was brought up by both patients and caregiver representatives but with slightly different meaning. Patients were concerned with unnecessary waiting time. One of the patients at the workshop concluded that “Making contact over phone takes time.” Being put on hold or needing to elaborate with work hours to visit the primary care unit can be seen as compromising with an individual’s time.

From a caregiver perspective, there is a clear concern about being efficient with the limited economical resources that are available. The high costs associated with treating T2D and other chronic illnesses became clear in the interviews with the management representative who expressed the importance of finding efficient work processes and right resources being allocated to the right needs. In the current work practice, the diabetes specialist nurse faces the challenge of limited resources alone as the diabetes care is mostly organized around the nurse and to a lesser extent the physician responsible for diabetes care at the unit. The interviews revealed that there were ambitions toward dealing with this issue through more developed teamwork at the primary care unit. Through involving more professions at the unit, for example, dietitians and physiotherapists, the individual needs of the patient are expected to be met in a better way. Moreover, the diabetes specialist nurses attending the workshop also expressed a similar concern about resource allocation, saying that if resources were distributed wisely, there would have been “More time for those who need it more.”

### Reduced Administrative Tasks

Seen from a caregiver perspective, administrative tasks are seen as a source of frustration. The observations and the interviews at the primary care unit revealed a work process surrounded with administrative, sometimes manual, tasks. The overall experience is that they are forced to prioritize among administrative tasks. Much of the work is also governed by an increased use of checklists that takes up time from other tasks. The IT support is, however, poor. For example, there is currently no automated system support for the diabetes specialist nurse to schedule appointments for patients for their biannual visits at the primary care unit. Instead, this is a cumbersome process, keeping track of the list of patients when it is time for the next visit, and there is always a risk of forgetting a patient or even that a patient gets accidently delisted. Making appointments can also include managing other resources at the primary care unit, for example, when a patient needs to take blood samples in advance and, therefore, needs to visit the laboratory unit. After a patient consultation, there are also the necessary tasks of reporting the visit in the electronic patient record and making notes in the Swedish National Diabetes Register. There is no integration between these two systems, resulting in registering the same data at two places.

**Table 2:** Themes and subthemes over challenges for design of a person-centered electronic health service for type 2 diabetes self-management support.

Themes	Subthemes
Improved access	Increased number of contact channels; access governed by needs
Resource efficiency	Facing limited resources; developed teamwork
Reduced administrative tasks	Prioritizing among administrative tasks; increased use of checklists
Continuous training and support	Responding to lifelong learning needs; relevant information technology training
Tailored care	Assessing patients’ individual needs; practicing person-centered care
Strengthened communication	Avoiding one-sided communication; counteracting shallow patient interaction
Patient empowerment	Improved responsibility and self-care; striving for disease control and balanced life; promoting independency

### Continuous Training and Support

Technical problems and complicated interfaces are a reoccurring concern among both patients and health care personnel. Although participants at the workshop see a great potential in more digital support, there is also a fear that this might be a complicated and cumbersome transition. One of the patients during the workshop questions if the eHealth service will be “Hard to handle, hard to learn?”. Similar concerns can be seen throughout the workshop participants, independent of background. There is an awareness that T2D patients are a diverse user group and that there are older people and people without much experience of digital tools. What is wanted is something that is “simple to use *”* and comes with “simple support *,”* thus responding to lifelong learning needs and accounting for differences in skill and previous knowledge.

Another problem related to learning new systems was revealed in the interviews at the primary care unit. Training is seen as important, but there are also expectations for it to be relevant. However, training is often given through Web-based course packages where the user is supposed to watch instruction videos. The videos are often long, and it is, therefore, hard to find suitable time for sitting down and watching during an ordinary workday. Due to these obstacles, there is a great risk that the training will be fragmented or even ignored. There is also a lack of opportunities for revised and more in-depth training for experienced users.

### Tailored Care

The workshop revealed a strong wish and expectation for more individualization when using eHealth solutions. A patient emphasized that “I am unique! Individual treatment.” There was an overall awareness among the participants that T2D was an individual experience, and there was no *one-size-fits-all* solution for everyone. Patients clearly express the need for more personalized treatment and alertness for individual needs.

Caregivers shared the patients’ aim for more personalization and expressed an expectation that the eHealth technology should provide better tools for assessing patients’ individual needs. A nurse pointed out that it would be good with a “Tailored profile, what the patient should work towards.” Availability of more and more easily accessible data was brought up as a promising enabler. This orientation toward personalization can also be seen in the interviews at the primary care unit, with the active aim toward practicing PCC through new routines and by including more professions into the T2D care based on patients’ individual needs.

### Strengthened Communication

Observations at the primary care unit showed that nurse-patient communication had a tendency to be one-sided. The nurse was leading the conversation, and the patient was passively listening and responding to direct questions. In one of the interviews, the specialist diabetes nurse commented on this and pointed out that it was hard to enable an open dialogue during these consultations. There are many things to go through during the visit, such as reviewing test results and medication, and there is, therefore, limited time for more open discussions. However, the nurse also pointed out that this varied depending on the individual patient. A patient with good self-management skills requires less time for standard activities, which leaves more time for dialogue.

The problem with communication was also brought up during the workshop. The caregiver representatives had high hopes for future eHealth solutions, and one of the nurses expressed that this could provide “more efficient communication which will save both time and money for patients and caregivers.” However, from the caregiver perspective, the main objective for better communication seems to be connected to resource efficiency. The patient representatives at the workshop expressed a different perspective on communication, suggesting that a change of perspective might help avoid one-sided communication. This required more attention to issues that are important for them. One of the patients stated that it was important to “speak about feelings, fears and anxiety,” which stood in strong contrast to the focus on practical checkpoints, as revealed in the observations.

### Patient Empowerment

Throughout the workshop, the participating nurses expressed a wish that future eHealth services would help patients improve responsibility and their self-care capability. Their expectations are that an eHealth service will provide patients with tools for being more engaged in their disease management and that increased involvement will promote increased independency among the patients. Moreover, highlighting the importance of involving relatives, one of the nurses said that the aim should be to “make oneself redundant—to be able to work in a way that makes patients and relatives flourish.” More involvement and independence were not exclusive for the caregiver perspective. The patients participating in the workshop had similar aims, expressing a strive for disease control and balance in life, and this could be achieved through eHealth services that supported independent and engaged self-management. One of the patients expressed the aim “to be able to handle ‘everything’ on my own without help, make everyday life easier.”

## Discussion

### Emergent Design Challenges

The 7 themes presented in the Results section were further discussed and abstracted by the authors and found to be bridged by three overarching concepts representing emergent design challenges. Challenges similar to these are suggested to have a large impact on the final design, and depending on the decisions made, one group of stakeholders might be favored at the expense of another group.

### Flexible Access: Critical Changes in Work Organization

Better and easier access to care was a prominent wish among patients. This can be supported in a new eHealth service, for example, through technology-enhanced communication such as instant messaging and video calls [34-36], thus offering an increased number of contact channels, and can also be achieved by changing current work processes at the primary care unit by making it easier for the patients to get in contact with the diabetes specialist nurse when needed. From a person-centered perspective, this would likely enhance the partnership between patient and nurse and support better physical and psychosocial well-being [17] for the patients by avoiding that small, but important, concern of being neglected.

However, promoting patients’ access to care will have implications for health care personnel. Easier access and more direct ways of communication require changes in the current work process to handle patient contacts. Today’s work practice involves gatekeeping patient contacts to support easier work planning and changing toward a more patient-initiated access will require primary care units to allocate resources to handle patient requests. This could also result in higher costs as some overstaffing can be required to handle unexpected peaks in the process flow.

We argue that to properly and fully address the person-centered perspective in design, the needs of the patient must come first. As pointed out, this will result in challenges for the health care organization when it comes to resource allocation; economy; and, which was evident in the results, resource efficiency. Overall, this can be seen as frustration with the current situation, when available resources do not match the actual need.

It could, however, be possible to find efficient ways of meeting patient needs through digital technology that does not necessarily require the patient to contact a person at the primary care unit. If we, for example, could better adapt the eHealth service to anticipate and react to user (patient) needs, for example, through data mining technologies [37], and automatically respond accordingly [38], some of the direct interaction with the primary unit could be avoided. This would benefit both the patient, through quick and accurate support, and the primary care unit that needs to allocate fewer, and also more appropriate, resources to this activity. However, to further explore this possibility, we need to pay closer attention to the everyday experience of the patients to better understand when critical questions occur and how we can respond. This includes gaining better understanding of the types of communication that have to be strengthened.

### Reducing Administrative Tasks: A New Division of Labor

In the Result section, administrative work and efficiency were mainly brought up by the diabetes specialist nurses and other stakeholders associated with the health care system. Given the current practice with many different IT systems and sometimes the necessity of registering the same data several times into different databases and records, it is easy to sense the frustration. Bringing up the idea of a new eHealth service brings forth anxiety that new systems equal more administration, and it is therefore brought up as a concern (cf [19]). Designing for this need would require close attention to current practices and routines and adequate support through automation. Moreover, integration between the new and existing systems should be provided.

The aspect of administration is, however, not well represented among the patients. There are concerns about avoiding unnecessary tasks and wasting time (eg, waiting on hold) but not to the extent of what is expressed by the health care personnel. We argue that this is not a sign of unimportance but rather that the participants lack a relevant point of reference to formulate an opinion. Introducing a new IT system (eg, an eHealth service) into an organization will, in almost all situations, affect work organization and the division of labor [39]. How people work and who is doing what work tasks will, intended or not, change. The practical implication of this will be the continuous training and support that were an expressed need among the health care personnel. To cope with the changes that new IT brings to an organization, users need to be trained properly to both being able to interact with the new system and getting accustomed to changing work tasks and processes. In the end, on an organizational level, this becomes a question of management, where work tasks can be distributed to a new group of workers, for example, to unlock other important resources. Therefore, when designing the organization, this becomes a clear and important delimiter.

However, when bringing in the person-centered perspective, the organization does not work as a delimiter of use. The new system is brought into a context comprising both the health care organization and the everyday life experience of the patients using it. This might result in a situation where tasks normally performed by nurses and other health care personnel are redistributed to the patient. More, and perceived unnecessary, work adds to the complexity of using the design and goes against the basic design principles of accessibility and usability [40] and might become a barrier for using the system, implying an increased need for relevant training and support also for patients.

We argue that this reduction of administrative tasks should be carefully considered in light of this extended context of use. This also creates a venue for asking questions about what patients consider as administrative tasks and what will be accepted. Again, this calls for closer attention to the everyday life experience of patients to better understand the potential impact of a new eHealth system and what administrative tasks can be acceptable.

### Patient Empowerment: Roles Are Changing

The results show that patients want independence and that the diabetes specialist nurses express that they want to support the patient in being more self-sustained. This is, however, not reflected in the current work process. Care is structured in such a way that it does not support independence, and the consultations mainly revolve around control; checking patient laboratory results; and the nurse leading the discussion, overall making the patient passive.

From a design perspective with focus on person-centered principles, a new eHealth service must focus on supporting patients and strengthening nurses in supporting the patients. Following the guidelines suggested by Wildevuur et al [17], this would include designing for shared decision making, mainly through enhancing communication and strengthening the partnership. Critical design decisions will have to be made that have a great impact on the role of the diabetes specialist nurse. The new eHealth service, if supporting person-centered perspectives and patient empowerment, will require the health care organization to initiate substantial changes in the organization, and the diabetes specialist nurses will have to adapt to this change and fine-tune their own work practice accordingly. This accommodates the expressed need for more tailored care, in which the nurse takes on a coaching role providing individualized support to the patient [14]. In addition, this would include a change in communication patterns, inviting the patient into a more in-depth interaction with the diabetes specialist nurse.

Implementing digital technology, for example, through a new eHealth service, will often have impact on power structures [39]. We argue that these power structures must receive closer attention and that the potential change of roles [41,42] when designing person-centered eHealth services for T2D self-management should also be implemented. Digital technology has the potential to either help in restructuring power, that is, changing the roles, or act to preserve existing structures. Proper design decisions have to be made to achieve the wanted effects and with an awareness and readiness that this will have a substantial impact on work organization at the primary care unit.

### Methodological Discussion

The methods used performed well in highlighting user needs from different perspectives and supporting an understanding of the current context and practice. It also worked well with the intended focus on patient-nurse interaction and the challenges that emerged. It does, however, not shed sufficient light on patient’s everyday experience and is, therefore, not enough for fully establishing the user needs for the patient group. To fully cover the patient perspective, the method needs to be adapted for that specific context.

### Conclusions

The aim of this study was to explore challenges that could impact the design of a person-centered eHealth service for T2D self-management. The results highlighted challenges or areas of concern that were seen as important and in which critical decisions have to be made. These challenges greatly affect both patients and health care personnel (diabetes specialist nurses in particular) and are essential points to be accounted for when designing a new eHealth service. To design in line with person-centered principles, the patient perspective needs to be favored, which in turn will have an impact on how work is organized and implemented at the primary care unit. Technology could possibly mitigate some of the impact on the organization, but to avoid a preponderance toward a primary care perspective, this would require more insights on how patients should be supported in everyday life, implying the use of other methods for exploring that particular context of use. This requires further research as it is not covered in this study.

## Abbreviations

eHealth: electronic health
IT: information technology
PCC: person-centered care
T2D: type 2 diabetes
UCD: user-centered design
